# Universal access: the benefits and challenges in bringing integrated HIV care to isolated and conflict affected populations in the Republic of Congo

**DOI:** 10.1186/1752-1505-3-1

**Published:** 2009-01-07

**Authors:** Daniel P O'Brien, Clair Mills, Catherine Hamel, Nathan Ford, Kevin Pottie

**Affiliations:** 1 Médecins Sans Frontières Holland, Amsterdam, The Netherlands; 2 Médecins Sans Frontières-Holland, Brazzaville, Republic of Congo; 3 Médecins Sans Frontières South Africa, Cape Town, South Africa; 4 Centre for Global Health, Institute of Population Health and Elisabeth Bruyère Research Institute, University of Ottawa, Ottowa, Ontario, Canada

## Abstract

The Pool region of the Republic of Congo is an isolated, conflict-affected area with under-resourced and poorly functioning health care services. Despite significant AIDS-related mortality and morbidity in this area, and a national level commitment to universal HIV care, HIV has been largely neglected. In 2005 Médecins Sans Frontières decided to introduce HIV care activities. However, in this setting of high basic health care needs, limited medical resources and competing medical priorities, a vertical HIV programme was not suitable. This paper describes the process of integrating HIV care and treatment into basic health services, the clinical outcomes of 222 patients started on antiretroviral treatment (ART), and the benefits to communities and health care systems. Key lessons learned include the use of multi-skilled human resources, the step-wise implementation of HIV activities, the initial engagement of an HIV experienced staff member, the use of simplified and adapted testing, clinical and monitoring protocols and drug regimens, the introduction of more complex monitoring tools to simplify clinical management decisions and intensive staff education regarding the benefits of HIV integration. This project in a rural and remote conflict-affected setting demonstrates that integrated HIV programs can save lives and play a key role in helping to achieve universal access to ART in Africa.

## Background

The Republic of Congo (RoC), situated in central Africa, has 3.8 million inhabitants [1] of whom about 70% live in the cities of Brazzaville and Pointe-Noire. RoC is rich in natural resources (e.g. petroleum and natural gas, timber, minerals, hydro-power). It was one of the most developed sub-Saharan African countries in the early 1980s, but began to decline by the end of that decade, the situation exacerbated by three civil wars between 1993 and 1999 and further civil conflict in 2002–3. A ceasefire was signed in March 2003, but fighting has continued in some areas. Corruption, arms spending and excessive borrowing against future oil production has left the country with one of the largest per-capita debts in the world.

The RoC health system operates using a cost recovery mechanism where patients pay a significant proportion of the care costs (e.g. around 2–3 EUR for a consultation and 30 EUR for a caesarean section). Access to health care is generally poor, either due to geographic distance or cost of services, leading many people to turn to traditional healers. Most health services outside the main cities are poorly staffed and lack basic drugs and equipment. In RoC life expectancy is 54 years, with an infant mortality rate of 108 deaths/1,000 live births [2]. The leading causes of morbidity and mortality are malaria (30–38%), respiratory tract infections (15–22%) and diarrhoeal diseases (7–15%). Tuberculosis (TB) is common (estimated incidence; 377/100,000 pop/yr) [3] and the reported prevalence of HIV is 4.2%. [4]

The 'Pool' region of RoC was significantly affected during the 1998 and 2002 civil wars by fighting between three political factions and their accompanying militias (Ninjas, Cocoyes and Cobras). Pool is one of nine departments in RoC, and was known for its serenity and natural beauty before the war; today it is a devastated area. Between 2003–2008 Médecins Sans Frontières (MSF) supported the hospitals and surrounding health centres in the administrative centre of Kinkala and 2 other towns Mindouli and Kindamba. Roads were very poor: the 90 km trip from Brazzaville to Kinkala which took one hour before the war, took over six hours in 2005 when the HIV project was started; Mindouli (70 km further) took another 4 hours. Security incidents on the road were common, and during the wet season the roads at times were impassable. Since 2006 the security situation in the region improved allowing the local economy to grow, but access remains difficult.

Kinkala hospital is an 80-bed referral hospital for the western part of the Pool region for an estimated population of 30,000. Mindouli hospital is a regional, 60-bed hospital serving a population of around 50,000. MSF's support to these hospitals comprised general medical, surgical, maternity, paediatric and mental health care (war trauma counselling), as well as nutritional and TB interventions. All health care services were provided free of charge.

Up to 2005 there was no capacity for HIV care in the region, and antiretroviral care was not available outside of the main urban centers of Brazzaville and Pointe Noire (where access was limited due to user fees). In addition, there was minimal knowledge of HIV and its treatment among health staff and the local population. However, health services in Kinkala and Mindouli were faced with large numbers of patients presenting to the health-care facilities with significant mortality and morbidity from HIV related illnesses, especially TB. Antiretroviral treatment (ART) programmes in developing countries, particularly in sub-Saharan Africa, have mainly been delivered through vertical programmes [5,6]. However in this rural and remote setting, with high basic medical needs and limited resources, it was considered that a vertical approach was unsuitable. Instead, a programme was established to offer HIV services as part of existing health services.

## Design of the integrated HIV care activities (Appendix 1)

### A. Programme management

The program focused on providing care to those presenting to the MSF or Ministry of Health (MoH) health facilities. HIV counseling and testing (CT) was targeted to those with an increased likelihood of having HIV and where knowledge of HIV would have an impact on the medical care provided: medical hospital inpatients, severely malnourished children not responding to treatment, patients with TB, sexually transmitted infections or illnesses suggestive of HIV.

A "Clinic for Chronic Diseases" was opened to provide care for patients with HIV as well as a number of chronic conditions including diabetes, epilepsy and hypertension. This was with the dual purpose of providing chronic disease management and limiting possible stigma associated with an "HIV clinic". [7] Nevertheless, in reality most patients had HIV (and/or TB). The clinics were initially open for only two afternoons a week, but as the number of patients grew they opened on a daily basis. The patient files and HIV-related medications (ARVs and drugs for prophylaxis of opportunistic infections (OIs)) were kept securely in the clinic, but other drugs were obtained from the ward or pharmacy if required.

Rather than wait until everything was in place before starting, the HIV activities were added in a step-wise manner. Initially HIV education and CT were introduced, followed by treatment and prophylaxis of OIs, and eventually ART. In this way patients could benefit from the interventions that logically precede the others whilst allowing for the time and experience required for programme staff to introduce the other activities. Although a cost recovery system operated in RoC, and all other available HIV care in the country required patient co-payments, it was negotiated with the MoH that all HIV services would be provided for free – including consultations, medications, laboratory investigations and nutrition – given the negative impact of user fees on HIV programmes [5].

At the time HIV activities were introduced, the region was still classified as a conflict area, with no formal peace agreement signed between the parties. In January 2006, prior to the introduction of HIV activities, the expatriate team were evacuated from Mindouli for 2 weeks due to security issues. Based on experience from other conflict settings, [8] a plan was put in place to deal with the potential of program disruption to minimize the risk that patients would have to interrupt their ART, and thus allow them to access care without greatly endangering their future treatment. Despite the potential for disruption, the program did not suffer from interruption in the three years since ART commenced.

### B. Human Resources and training

Two extra doctors and two laboratory technicians were added to the health program to support the introduction of HIV activities. However it was also realised the single medical doctor (MD) previously in the program had been overworked and that HIV had 'justified' the addition of a resource that was already required prior to the addition of HIV activities.

The HIV component was included as part of staff members routine activities rather than having wholly dedicated "HIV" staff. Doctors caring for HIV patients also worked in the adult medical, paediatric, emergency and TB wards, and counsellors undertook general psychosocial counselling for HIV negative people (e.g. post-traumatic counselling) as well as HIV related counselling and education activities. Nevertheless, one MD was made chiefly responsible for HIV activities including direct clinical care.

Initially the health staff (expatriate and national) had no significant HIV care experience, and felt reluctant and fearful to begin. Thus to help plan and commence HIV activities, an MD with experience in treating HIV in resource-limited settings initially provided support in the project by designing care pathways, training staff, clinical mentoring and setting up data collection systems. Staff were also provided with short (i.e.1–2 weeks) practical experience in other large regional HIV programs (e.g. MSF in Kinshasa, DRC; French Red Cross in Brazzaville, RoC), and attending local or external courses.

A strong focus was placed on HIV education and awareness for all health, hospital and MSF program staff through general staff meetings and targeted training sessions. Space was given to discuss misconceptions, stigma and anxieties of health staff related to HIV, and to actively address these through ongoing regular education sessions.

One of the key successes of the integration process was the institution of regular meetings between counsellors, nurses, doctors and all others involved in the HIV/AIDS activities. These were used to discuss difficult patient cases, for education and training, and to share information, but they also served as an opportunity for supervisors to identify misconceptions or negative attitudes among staff. In addition, they helped create a cohesive interdisciplinary team approach to HIV activities which facilitated the implementation of the program across the various health activities.

### C. Clinical care

For diagnosis, HIV rapid diagnostic tests (Determine HIV-1/2^® ^and Unigold HIV^®^) were used on venous blood samples. Testing was done confidentially by laboratory staff, mainly because national regulations prohibit testing by non-medical staff. For ART, generic antiretroviral drugs were used in the form of fixed-dose combinations (FDCs) which facilitated adherence, procurement and stock management, and reduced costs. Eligibility criteria for ART and first-line regimens were standardised and based on WHO recommendations [9]. During the initial phase only first-line drugs and their alternatives were provided (i.e. stavudine, lamivudine, nevirapine, effavirenz, zidovudine and nelfinavir) to allow simplification, based on the fact that almost all patients were ART naïve and thus not likely to need second-line ART for at least 12 months [10]. For treatment of OIs, the simplest effective protocols were used (e.g. fluconazole rather than intravenous amphotericin B for initial treatment of cryptococcosis, and cotrimoxazole rather than sulphadiazine and pyrimethamine for treatment of cerebral toxoplasmosis). Clinical consultations were performed by both doctors and nurses. Monitoring was performed on a clinical and immunological basis (CD4 count) with no viral load monitoring.

It has been argued that monitoring tools such as CD4 machines are too complex for many resource-limited settings. [11] However we found that a simplification of management was achieved by instituting some 'complex' monitoring tools such as CD4 counts and liver function tests that increased the ease of decision-making by less experienced clinical staff, a process we would describe as 'paradoxical simplification'. Clinical staff found HIV management (e.g. initiation of treatment and prophylaxis, or monitoring effectiveness of treatment) easier if they had a 'number' to follow rather than having to rely on clinical assessments alone. A Sysemex machine capable of performing automated CD4 counts was introduced into the Mindouli laboratory. In the same logic, an automated biochemistry machine was installed to support the monitoring and management of ART related side-effects (e.g. hepatitis, renal dysfunction), which also increased hospital capacity for diagnosis and management of other non-HIV related medical conditions.

In a population with little knowledge of HIV or the benefits of treatment, it was felt that strong efforts were needed to encourage patients to commit fully to ART. Before starting ART patients were required to attend at least 2–3 education and adherence workshops (usually in groups) on HIV and how ART medications work, common side effects and how to overcome them, the importance of adherence, and drug resistance. Counselors had a strong input into decisions regarding a patient's readiness to commence ART, including an assessment of the patient's understanding of the disease and ability to take ART. A patient support group was also created which was facilitated by the counsellors with the presence of a doctor or nurse on occasions.

## HIV activities and outcomes

HIV activities began with HIV counseling and testing in Kinkala in March 2005 and in Mindouli in February 2006. All HIV activities were transferred from Kinkala to Mindouli in May 2006 as MSF withdrew its support in Kinkala, and in Mindouli were handed over to the MoH in February 2008.

Overall, 1058 HIV tests were performed of which 388 (37%) were positive. Of those HIV positive, 352 (91%) accepted medical care; 95% were ≥ 15 years of age and 71% were female. By the end of December 2007, 236 (76% of those in medical care) people had commenced ART in the Kinkala/Mindouli program; 222 (94%) adults and 12 (5%) children<15 years of age (age unknown for 2 people).

Baseline characteristics and outcomes for adults are shown in Table 1. By end 2007, the mean duration on ART was 9 months. There were 20 (9%) deaths occurring after a median 2.2 months (IQR 1.2–10.7 months) on ART; 65% within the first 3 months. Twenty-nine (13%) were lost to follow-up after a median of 4.1 months on ART (IQR 1.2–7.4). Survival probabilities, immunological and clinical outcomes were good and in keeping with cohorts in other African settings (Table 2)[5,6]. No children on ART died or were lost to follow-up after a median of 7 months on ART.

**Table 1:** Characteristics at ART baseline: Adults

Total number of patients	222
Median age [IQR] (years)	37.0 [32.0–43.0]
Female (%)	153 (68.9)
BMI (kg/m^2^): N	205
Median [IQR]	17.9 [16.4–19.4]
< 17 : n (%)	70 (34.2)
17–18.4 : n (%)	54 (26.3)
≥ 18.5 : n (%)	81 (39.5)
CD4 done at initiation^1^: N	176
Median CD4 count [IQR]	104.0 [39.5–172.0]
WHO clinical stage: N	210
Stage 4 (%)	92 (43.8)
Stage 3 (%)	112 (53.3)
Stage 1/2 (%)	6 (2.9)
ART naïve^2^: n (%)	211 (95.0)
Initial ART Regimen: N	222
3TC+D4T+NVP	168 (75.7)
3TC+D4T+EFV	32 (14.4)
Other	22 (9.9)

^1 ^CD4 obtained between 3 months before and 1 month after ART are taken into account

^2 ^Women who, before ART initiation, took PMTCT ARVs only are considered as naive

IQR : interquartile range.

**Table 2:** outcomes on ART for adults

	N	Result
Probabilities of survival^1 ^(95%CI)		
at 6 months	129	0.94 [0.89–0.96]
at 1 year	70	0.89 [0.82–0.93]
New WHO clinical stage 3 or 4 events n (%)		
between 0 and 12 months	222	72 (32.4%)
between 1 & 2 years	70	10 (14.3%)
Median CD4 (in cells/mm^3^)		
at 6 months	53	205.0
at 1 year	29	202.0
Median CD4 gain (in cells/mm^3^)		
at 6 months	32	91.0
at 1 year	25	104.0
BMI < 17 n (%)		
at 6 months	105	14 (13.3%)
at 1 year	52	6 (11.5%)

1 Combined endpoint of those not dead or lost to follow-up

Thus ART has been commenced for a significant number of patients (especially for an integrated rural-based program) and the outcomes have been good with important individual benefits.

## Challenges in implementation of integrated HIV/AIDS programs (Appendix 2)

One of the greatest challenges was to convince staff (both expatriate and national) working under basic conditions with high medical needs and limited resources of the need and capability to introduce HIV care. It was essential to promote and/or develop a sense of ownership and motivation within the field teams to make HIV integration work. Some staff felt that there were greater medical priorities; malaria, diarrhea, respiratory illnesses, malnutrition and maternal and infant health. Staff were also unfamiliar and uncomfortable with HIV management and there was a fear that HIV care, through its perceived complexity and time demands on already overworked staff, would turn the focus of care too much towards HIV and detract from the ability to provide for these other needs. Concerns were addressed through education and discussions, including explaining that although there may appear to be higher priority health care needs, most of the major morbidities confronted occur more frequently and have a higher mortality in the presence of underlying HIV. Thus addressing HIV would substantially contribute to addressing these other health care needs. In addition, the introduction of HIV activities allowed the justification to program managers of extra resources that were in fact already needed (e.g. an extra doctor, extra laboratory resources). Thus rather than draining resources from other services, staff discovered that introducing HIV care led to a strengthening of medical activities in other areas. Overall, the experience was that introducing HIV services led to minimal disruption to other activities while providing additional, much needed resources.

One of the challenges of introducing HIV care into an area with minimal HIV knowledge or awareness is a concern over stigma and negative consequences from health staff, families and community for those diagnosed as HIV positive[12]. This may lead to excessive confidentiality measures being instituted that lead to secrecy rather than appropriate practice. Early in this program medical practices that were potentially dangerous were instituted such as not writing patient's HIV drugs on the medication chart or recording their HIV status in their medical history, and not informing health staff providing direct patient care – for instance the TB nurse – of a patient's HIV status. To overcome this, an approach was needed where openness around HIV testing and treatment was promoted. For example that it is normal and beneficial for people to know their HIV status and that all people for whom HIV infection would complicate their illness should be encouraged to be tested (e.g. all patients on medical, TB and therapeutic feeding wards were given group counselling and offered HIV testing). In addition normal codes of medical confidentiality were instituted with efforts to ensure that HIV results and medications were entered into the medical files and drug charts, and that a patient's HIV status was appropriately shared by staff caring for patients.

As care was targeted towards patient groups with high levels of immunosuppression (e.g. medical inpatients, those with symptoms) and TB co-infected patients, teams were confronted with the significant early mortality rates on ART frequently described from African programs[5], especially in the early phases of integration. This had the effect of initially reducing the confidence of the inexperienced medical staff in managing ART, and reinforced fears around safety of ART amongst patients. In this situation, efforts were required to reassure staff and patients of the reasons for the high mortality, and to promote initiation of treatment for eligible but asymptomatic patients to simplify patient management and demonstrate success.

In a region where both patients and staff were unfamiliar with HIV, there were many debates within health staff regarding the 'ethics' of offering HIV testing, especially with the lack of guaranteed long-term availability of ART. For example, there were understandable concerns that people tested for HIV could face serious negative consequences if tested positive (e.g. abandonment, physical violence, discrimination), and people were often not convinced that the benefits of testing outweighed these concerns. Thus teams were often reluctant or actively opposed to offering CT; this was most evidenced by a reluctance to offer HIV testing in the antenatal clinic for mainly asymptomatic pregnant women. To address these concerns, careful and repeated discussions from staff experienced in HIV management outlining the benefits and the means of minimising the risks of testing were required.

## Benefits of introducing HIV/AIDS activities into medical programs

### 'Towards Universal Access'

While there is clear international consensus to provide universal access to HIV care [13], most programs in resource-limited settings have to date been vertical programs in urban areas [5,6]. Integrated programs have the potential to allow HIV care to be provided in an increasing number of programs and to more rural populations. The program in the Pool region provided care to a very disadvantaged population: rural, poor, isolated and conflict-affected.

### Combating stigma and increasing HIV awareness

HIV-related stigma significantly impacts on uptake of HIV testing, and adherence to HIV treatment and follow-up[14]. In this program it was experienced that as increasing numbers of patients in the program benefited from care, going from poor health to living full lives on ART, the level of HIV-related stigma amongst health staff and the community decreased and likely contributed to increased uptake of HIV testing and care.

### Building staff morale and program cohesiveness

Despite the extra workload, most health staff found it a very positive experience to be able to offer treatment to patients with HIV, develop skills in managing HIV and witness the life-saving effects of ART. In addition, the integration of activities facilitated the cohesiveness of previously existing medical activities (e.g. bringing psychosocial, nutritional and medical services closer together).

### Capacity building

Vertical HIV programmes have been criticized for their potentially harmful effects of draining resources from basic health services [15]. However this program was successful in significantly increasing the capacity of the local health infrastructure to deal effectively with HIV/AIDS as well as other illnesses. This occurred by increasing the knowledge and motivation of health staff, introducing quality patient management care systems, supporting the development of robust drug monitoring and procurement systems, introducing standard data collection processes, identification of increased funding for staff and materials, and the introduction of more sophisticated laboratory equipment.

### Catalyst for engagement

The introduction of HIV care, initially by an international NGO, acted as a catalyst for the MoH and other actors to engage and commit to HIV in the region. In a national program struggling to implement care outside of the main urban centers, a difficult to access, potentially dangerous and neglected area like the Pool was not high on the priority list to commence treatment programs. However by 2008 the RoC National AIDS control program (NACP) had included Kinkala and Mindouli as ART treatment centers.

### Creation of community advocacy groups

One of the added benefits of introducing HIV care is that people benefiting from treatment have a self-interest in ensuring that it remains available. The programme was active in the development of a HIV positive support group whose aims included holding the MoH and NACP accountable for the availability and quality of the ongoing HIV program.

## Challenges for the future of the program

The integrated HIV programme in Pool is faced with a number of challenges. Most significantly, the numbers of people living with HIV (PLHA) accessing care will steadily increase, and this will place increasing demands on the costs and workload of HIV care. However, as most PLHA will become well on treatment, this workload will be compensated by reduced needs for hospitalization and palliative care of AIDS sufferers, particularly as access to care expands and more people start ART before they are sick.

Another challenge is the prevention of mother to child HIV transmission (PMTCT). These activities have not yet been introduced mainly due to a lack of motivation from the field teams related to a combination of failing to understand the importance and benefits of PMTCT, a fear of harm to women being diagnosed HIV positive, lack of experience with PMTCT, and busy workload. In addition, the number of children diagnosed and treated has been suboptimal due to inexperience and fear of testing and treating children with HIV, but also because of a lack of diagnostic tools and adapted medications for young children [16]. Designing effective PMTCT interventions and increasing the number of children diagnosed and treated for HIV, especially as infants, [17] is an urgent priority for the future.

Finally, as the cohort of patients on ART matures, there will be an increasing need for second-line ARVs for those failing treatment [10]. This will place increasing strains on the project in terms of cost, complexity and sustainability, and access to second-line ARVs will need to be made available through the NACP.

## Conclusion

Integrating HIV care activities into basic health programs in conflict affected areas is possible with good individual outcomes and benefits to communities and health care systems. Nevertheless there are many challenges and dilemmas in implementation. Our experience in RoC, which adds to the growing evidence that ART delivery is effective in conflict settings[8,18], has yielded a number of important lessons that could benefit actors considering similar interventions. Integrated HIV programs have a role to play in rural and remote settings where they have the potential to save lives and play a key role in helping to achieve universal access to ART in Africa.

## Competing interests

The authors declare that they have no competing interests.

## Authors' contributions

DOB, CM and CH contributed to the design and implementation of the program. DOB and CH contributed to the data collection and analysis. All the authors contributed to the concept, writing and editing of the manuscript.

## Appendix 1

### Factors supporting integration of HIV activities into routine programmes in resource-limited settings

- Engage an HIV experienced staff member to support the initial set-up of the program

- Convince staff of the need and capability to introduce HIV care (share success stories)

- Specific training and coaching to establish multi-skilled staff with HIV activities included as part of other clinical duties; this could include short (1–2 week) placements in nearby HIV programmes

- Consider a chronic disease clinic to share treatment approaches and reduce HIV-related stigma

- Regular HIV education and awareness activities for all health staff

- Targeted HIV testing of high-risk patient groups

- Use simplified and adapted testing, clinical and monitoring protocols and drug regimens

- Consider introduction of 'complex' monitoring tools that 'simplify' management (paradoxical simplification)

- Introduce HIV activities in a step-wise manner so patients benefit as soon as possible while staff prepare for the next steps

- Ensure inclusion of asymptomatic patients meeting the criteria for ART commencement to both simplify management and provide motivational 'success stories'

- Provide HIV care services free of charge

- Ensure regular team meetings for all staff involved in HIV activities.

## Appendix 2

### Challenges in implementation of integrated HIV/AIDS programs in conflict areas

- Convincing staff of the need and capability to introduce HIV care when faced with other medical priorities, low resources and heavy workloads.

- Avoiding the development of excessive secrecy around HIV management that can be created in an attempt to maintain confidentiality

- Reducing the early high death rate of patients on ART that occurs when the most immunosupressed patients are targeted

- Overcoming the concern of staff over the risk versus benefits of introducing HIV counseling and testing in conflict settings

## References

[B1] Food and agriculture indicators. http://www.fao.org/es/ess/compendium_2006/pdf/PRC_ESS_E.pdf.

[B2] Mortality country fact sheet 2006. http://www.who.int/whosis/mort/profiles/mort_afro_cog_congo.pdf.

[B3] TB country profile. http://www.afro.who.int/tb/country-profiles/congo.pdf.

[B4] Epidemiological Country Profile on HIV and AIDS. http://www.who.int/globalatlas/predefinedReports/EFS2008/short/EFSCountryProfiles2008_CG.pdf.

[B5] Braitstein P, Brinkhof MW, Dabis F, Schechter M, Boulle A, Miotti P, Wood R, Laurent C, Sprinz E, Seyler C, Bangsberg DR, Balestre E, Sterne JA, May M, Egger M, Antiretroviral Therapy in Lower Income Countries (ART-LINC) Collaboration, ART Cohort Collaboration (ART-CC) groups (2006). Mortality of HIV-1-infected patients in the first year of antiretroviral therapy: comparison between low-income and high-income countries. Lancet.

[B6] Akileswaran C, Lurie MN, Flanigan TP, Mayer KH (2005). Lessons learned from use of highly active antiretroviral therapy in Africa. Clin Infect Dis.

[B7] Janssens B, Van Damme W, Raleigh B, Gupta J, Khem S, Soy Ty K, Vun M, Ford N, Zachariah R (2007). Offering integrated care for HIV/AIDS, diabetes and hypertension within chronic disease clinics in Cambodia. Bulletin of the World Health Organization.

[B8] Culbert H, Tu D, O'Brien DP, Ellman T, Mills C, Ford N, Amisi T, Chan K, Venis S (2007). HIV treatment in a conflict setting: outcomes and experiences from Bukavu, Democratic Republic of the Congo. PLoS medicine.

[B9] World Health Organisation (2006). Antiretroviral therapy for adults and adolescents in resource-limited settings: towards universal access. Geneva.

[B10] Pujades-Rodriguez M, O'Brien D, Humblet P, Calmy A (2008). Second-line antiretroviral therapy in resource-limited settings: the experience of Medecins Sans Frontieres. AIDS (London, England).

[B11] Phillips AN, Pillay D, Miners AH, Bennett DE, Gilks CF, Lundgren JD (2008). Outcomes from monitoring of patients on antiretroviral therapy in resource-limited settings with viral load, CD4 cell count, or clinical observation alone: a computer simulation model. Lancet.

[B12] Mahajan AP, Sayles JN, Patel VA, Remien RH, Sawires SR, Ortiz DJ, Szekeres G, Coates TJ (2008). Stigma in the HIV/AIDS epidemic: a review of the literature and recommendations for the way forward. AIDS (London, England).

[B13] World Health Organisation (2008). Towards universal access: scaling-up priority HIV/AIDS interventions in the health sector: progress report 2008. Geneva.

[B14] Castro A, Farmer P (2005). Understanding and addressing AIDS-related stigma: from anthropological theory to clinical practice in Haiti. American journal of public health.

[B15] (2008). Does HIV/AIDS still require an exceptional response?. lancet Infectious Diseases.

[B16] O'Brien DP, Sauvageot D, Zachariah R, Humblet P (2006). In resource-limited settings good early outcomes can be achieved in children using adult fixed-dose combination antiretroviral therapy. AIDS (London, England).

[B17] Violari A, Cotton MF, Gibb DM, Babiker AG, Steyn J, Madhi SA, Jean-Philippe P, McIntyre JA (2008). Early antiretroviral therapy and mortality among HIV-infected infants. The New England journal of medicine.

[B18] Kiboneka A, Nyatia RJ, Nabiryo C, Olupot-Olupot P, Anema A, Cooper C, Mills E (2008). Pediatric HIV therapy in armed conflict. AIDS (London, England).

